# Prelabor rupture of membranes and the association with cerebral palsy in term born children: a national registry-based cohort study

**DOI:** 10.1186/s12884-020-2751-3

**Published:** 2020-01-31

**Authors:** Maren Mynarek, Solveig Bjellmo, Stian Lydersen, Kristin Melheim Strand, Jan Egil Afset, Guro L. Andersen, Torstein Vik

**Affiliations:** 1 Department of Clinical and Molecular Medicine, Norwegian University of Science and Technology, Faculty of Medicine and Health Sciences, PO Box 8905, NO-7491 Trondheim, Norway; 2 Department of Obstetrics and Gynecology, Helse More og Romsdal HF, Alesund, Norway; 3 Department of Mental Health, Regional Centre for Child and Youth Health and Child Welfare, PB 8905, MTFS, 7491 Trondheim, Norway; 4 Department of Obstetrics and Gynecology, St. Olavs Hospital, Trondheim University Hospital, Trondheim, Norway; 5 Department of Medical Microbiology, St. Olavs Hospital, Trondheim University Hospital, Trondheim, Norway; 6 Vestfold Hospital Trust, The Cerebral Palsy Register of Norway, PB 2168, 3103 Tønsberg, Norway

**Keywords:** Cerebral palsy, Prolonged interval, Prelabor rupture of membranes, Perinatal infection, Neonatal mortality, Stillbirth

## Abstract

**Background:**

Guidelines regarding management of prelabor rupture of membranes (PROM) at term vary between immediate induction and expectant management. A long interval between PROM and delivery increases the risk for perinatal infections. Severe perinatal infections are associated with excess risk for cerebral palsy (CP) and perinatal death. We investigated if increasing intervals between PROM and delivery were associated with perinatal death or CP.

**Methods:**

Eligible to participate in this population-based cohort-study were term born singletons without congenital malformations born in Norway during 1999–2009. Data was retrieved from the Medical Birth Registry of Norway (MBRN) and the Cerebral Palsy Register of Norway. In line with the registration in the MBRN, intervals between PROM and delivery of more than 24 h was defined as ‘prolonged’ and intervals between 12 and 24 h as ‘intermediate’. Outcomes were stillbirth, death during delivery, neonatal mortality and CP. Logistic regression was used to calculate odds ratio (OR) with 95% confidence intervals (CI) for adverse outcomes in children born after prolonged and intermediate intervals, compared with a reference group comprising all children born less than 12 h after PROM or without PROM.

**Results:**

Among 559,972 births, 34,759 children were born after intermediate and 30,332 were born after prolonged intervals. There was no association between increasing intervals and death during delivery or in the neonatal period, while the prevalence of stillbirths decreased with increasing intervals. Among children born after intermediate intervals 38 (0.11%) had CP, while among those born after prolonged intervals 46 (0.15%) had CP. Compared with the reference group, the OR for CP was 1.16 (CI; 0.83 to 1.61) after intermediate and 1.61 (CI; 1.19 to 2.18) after prolonged intervals. Adjusting for antenatal factors did not affect these associations. Among children with CP the proportion with diffuse cortical injury and basal ganglia pathology on cerebral MRI, consistent with hypoxic-ischemic injuries, increased with increasing intervals.

**Conclusion:**

Intervals between PROM and delivery of more than 24 h were associated with CP, but not with neonatal mortality or death during delivery. The inverse association with stillbirth is probably due to reverse causality.

## Background

Prelabour rupture of membranes (PROM) is defined as spontaneous membrane rupture before the onset of contractions, and occurs in 6–19% of pregnancies at term [1]. The appropriate management is controversial [2] and current national guidelines on how to handle PROM at term differ significantly. The National Institute for Health and Care Excellence in England recommend expectant management [3] since spontaneous labor will start within 24 h in 70–90% of cases [4, 5]. Norwegian guidelines suggest induction of labor 24 h after PROM if there is no sign of infection and the CTG is reassuring [6]. Other guidelines recommend induction of labor as soon as possible [2, 7]. In the latter recommendations, expectant management may still be considered acceptable in patients who decline induction of labor, provided the woman is appropriately counseled, and that the clinical condition of the woman and fetus is reassuring [2].

One concern of expectant management is that a long interval between PROM and delivery may increase the risk for perinatal infections in the offspring [8]. Such infections are associated with severe neonatal morbidity and increased risk of perinatal death. Among survivors, a potential long-term outcome may be cerebral palsy (CP) [9, 10]. A recent systematic review on risk factors for CP in term born children [10], identified only one article exploring the association between the interval from PROM to delivery, and CP [11]. That study found a 54% higher risk for CP in children who had been born more than 24 h after PROM. However, this excess risk did not reach statistical significance.

A Cochrane review concluded that current evidence in favor of immediate delivery following PROM was of low quality, and that more evidence was needed [7].

On this background, the primary aim of the present study was to investigate if the interval between PROM and delivery was associated with increased risk of CP. As secondary aims we wanted to explore if specific CP subtypes and/or specific cerebral MRI findings were associated with increasing intervals. Finally, we wanted to study if increasing intervals from PROM to delivery were associated with stillbirth, death during delivery and neonatal mortality.

## Material & methods

### Study design and population

Eligible to participate in this population-based cohort study, were all children born at term, (i.e. between 37.0 and 43.6 weeks of gestation) during 1999–2009. Children with missing information regarding timing of PROM, multiple births and children with congenital malformations were excluded, as well as children whose birth weight standard deviation scores exceeded 6 standard deviations from the mean after adjusting for sex and gestational age (GA). The decision to exclude children with a birth weight standard deviation score that exceeded 6 standard deviations, was arbitrarily taken, as such deviations in scores were most likely due to data recording errors.

Maternal, pregnancy and obstetric information of the current pregnancy was retrieved from The Medical Birth Registry of Norway (MBRN) and combined with information on CP recorded in The Cerebral Palsy Register of Norway (CPRN). Data from the two registers was linked using the 11-digit personal identification number unique for every Norwegian citizen.

The MBRN records information on all births after 12 weeks of gestation in Norway. The register comprises demographic information and information on various aspects of the pregnancy and birth, including maternal health, delivery and treatment of the newborn [12].

The CPRN is an informed consent based national quality register recording information on all children with CP born since 1996 [13]. It contains detailed clinical information including CP subtypes, fine and gross motor impairments and associated problems as well as an extract of the qualitative descriptions on cerebral MRI. The data is provided by pediatricians working at the public neuropediatric habilitation centers caring for all children with CP in Norway. Clinical information is reported to the register at the time of diagnosis, at 5 years of age when the diagnosis has been confirmed, and finally at 15–17 years of age. In this study we have used data from the five years’ registration, collected in 2016 in order to account for any delayed registration (diagnosis).

The main exposure variable was the interval between prelabour rupture of membranes (PROM) defined as rupture of membranes before the onset of contractions, and delivery of the child. In the MBRN, PROM is a unique variable and is recorded based upon the interval until delivery, as more than 24 h, or between 12 and 24 h. Intervals of shorter duration (i.e. less than 12 h) are not recorded as PROM in the MBRN.

We defined intervals of more than 24 h as ‘prolonged’ intervals and intervals between 12 and 24 as ‘intermediate’ intervals. The ‘reference group’ comprised children born less than 12 h after PROM and children born after deliveries where the membranes ruptured at onset of contractions or during the active phase of labor.

The primary outcome variable was CP, diagnosed and classified according to the definition recommended by the Surveillance of Cerebral Palsy in Europe as a group of permanent and non-progressive disorders of movement and posture caused by a central nervous lesion, damage or dysfunction originating early in life [14]. According to the SCPE CP is classified into the subtypes spastic, dyskinetic and ataxic based upon the predominant neurological finding and the spastic subtypes are further divided in bi- and unilateral spastic CP [14].

Secondary outcomes were stillbirth, death during delivery or in the neonatal period. Stillbirth is recorded in the MBRN in line with the definition proposed by the World Health Organization (WHO) as a baby born with no sign of life [15]. Stillbirth was further divided in death before or during birth. Neonatal mortality is defined as death during the first 28 days of life [15].

We also studied the association between the interval from PROM to delivery and CP-subtypes, gross- and fine motor function, speech ability and epilepsy. In a subgroup of children with CP who had been examined with cerebral MRI, we studied the association between the interval from PROM to delivery and MRI findings.

Gross motor function was classified into five levels according to the Gross Motor Function Classification System [16]. Fine motor function was also classified into five levels, using the Manual Ability Classification System [17]. In both classification systems, level I indicates the least severe and level V the most severe impairments. Speech ability was classified into four levels using the Viking speech scale, whereby level I indicates normal speech and level IV no understandable speech [18]. Epilepsy was defined as present in children with the diagnosis, who were treated with antiepileptic drugs. The presence of gastrostomy tube feeding was used to indicate severe eating difficulties.

The cerebral MRI examinations were obtained at different ages, and the results were recorded in the CPRN on the five years’ registration form. The responsible neuropediatrician reported if a cerebral MRI had been performed, the child’s age at the time of examination and provided an extract of the findings as described by the local radiologist.

Information on maternal age, parity, maternal diagnosis before and during pregnancy, specific diseases such as; diabetes mellitus, thyroid disease, recurrent urinary tract infection, chronic kidney disease, in vitro fertilization, fetal position, induction, cesarean section, sex, GA, birth weight and Apgar score was retrieved from the MBRN. GA was based upon ultrasound examination around pregnancy week 18 in 97% of the included births. In the remaining cases estimates were based upon the last menstrual period [19].

Newborns with a birth weight below − 2 standard deviations, adjusted for GA and sex according to Norwegian growth standards, were defined as being small for gestational age.

Birth weight standard deviation scores were calculated using a sex-specific reference for fetal growth by Skjaerven et al. [20]

### Statistical analysis

Chi square statistics were performed to explore differences in proportions between groups. To study trends, we used the linear-by-linear association test, or the Kruskal-Wallis test (for rxc tables with number of rows r > 2 and number of columns c > 2) and the Cochran-Armitage test for trend (for 2xc tables). Prevalence per 1000 with 95% confidence intervals (CI) was calculated using the Wilson score as recommended by Fagerland et al. [21] Logistic regression was used to calculate odds ratio (OR) with 95% CI as estimates of the relative risk for CP in children born after intermediate and prolonged intervals, compared with the reference group. Possible confounders of the association between prolonged intervals and the adverse outcomes were selected based upon theoretical considerations using the hierarchical approach proposed by Victora et al. [22] The following potential confounders were explored: maternal age, parity, medical diagnoses before and during pregnancy; specifically primary or gestational diabetes mellitus, thyroid disorders, recurrent urinary tract infection, in vitro fertilization, sex, gestational age, birth weight small for gestational age and fetal position. Variables changing the unadjusted OR more than 10% were considered potential confounders and were included in further analyses. *P*-values below 0.05 were considered to indicate statistical significance. Statistical analyses of the data were performed using IBM SPSS Statistics 24.

## Results

**Fig. 1:**
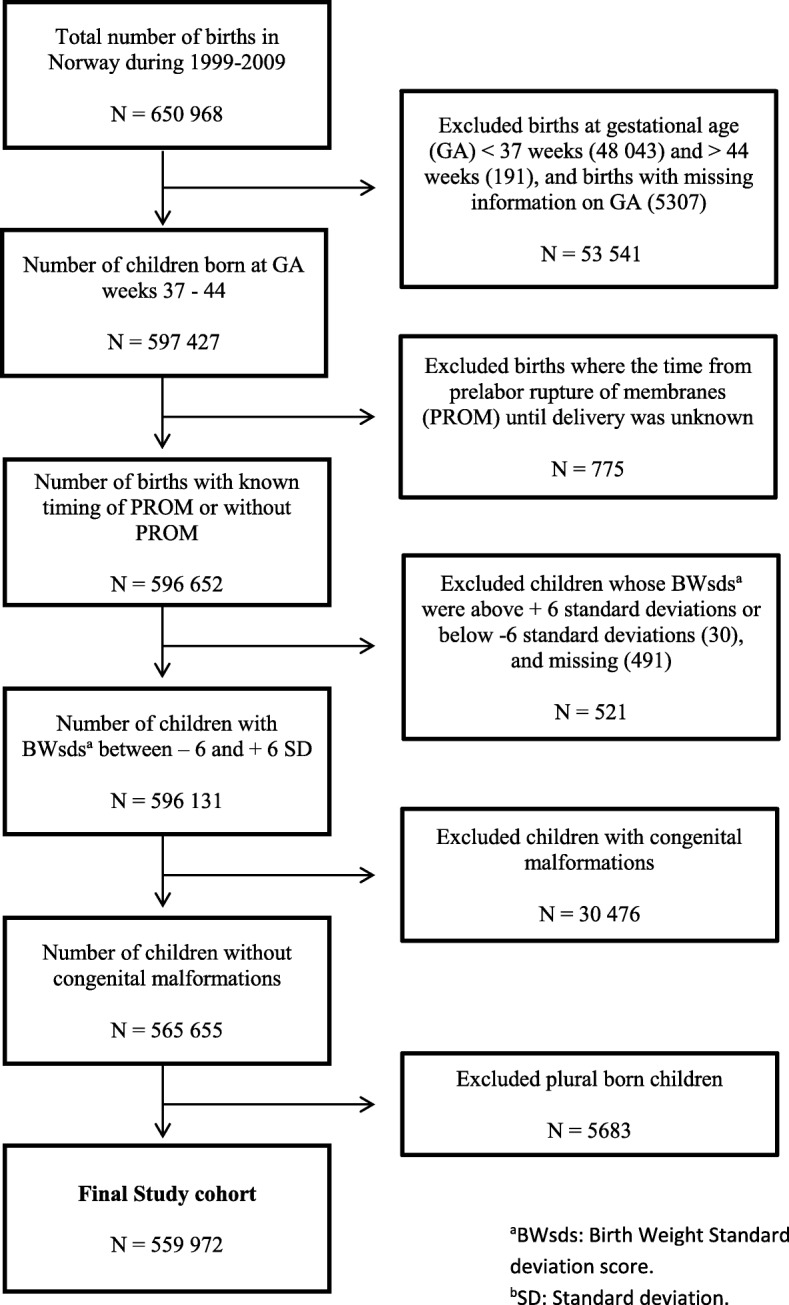
Flowchart of the study population

In total, 650,968 children were born in Norway during 1999–2009. The study population comprised 559,972 children after exclusion of children whose birth weight standard deviation score exceeded 6 standard deviations from the mean after adjusting for sex and GA, children with missing information regarding timing of PROM, multiple births and children with congenital malformations (Fig. 1).

A total of 494,881 (88%) were born in the reference group, 34,759 (6%) children were born after intermediate intervals between PROM and delivery, and 30,332 (6%) children after prolonged intervals. There were few differences in maternal and infant characteristics between the three groups (Table 1). As expected, the proportions of induced deliveries, as well as acute caesarean section increased with increasing intervals from PROM until delivery, while the proportion delivered with elective caesarean section decreased (Table 1).

**Table 1:** Maternal and infant characteristics according to the interval between prelabor rupture of membranes and delivery

	Reference group		Intermediate interval^a^		Prolonged interval^b^		
	N	(%)	N	(%)	N	(%)	
Number of births	*494,881*	*(100)*	*34,759*	*(100)*	*30,332*	*(100)*	*P-value*
Maternal age^e^							
< 19	11,681	(2.4)	784	(2.3)	623	(2.1)	
20–34	401,202	(81)	28,727	(83)	24,609	(81)	
> 35	81,977	(17)	5245	(15)	5097	(17)	0.55^c^
Parity							
Para 0	185,723	(38)	21,827	(63)	19,257	(64)	
> 0 Para	309,158	(63)	12,932	(37)	11,075	(37)	< 0.001^c^
Any maternal diagnosis before pregnancy	204,310	(41)	14,545	(42)	13,391	(44)	< 0.001^c^
Any maternal diagnosis during pregnancy	145,583	(29)	9959	(29)	8889	(29)	0.096^c^
Diabetes mellitus^f^							
Diagnosed before pregnancy	2671	(0.5)	221	(0.6)	164	(0.5)	
Gestational	4462	(0.9)	375	(1.1)	285	(0.9)	0.007^d^
Thyroid disease	6888	(1.4)	495	(1.4)	465	(1.5)	0.046^c^
Recurrent urinary tract infection	15,458	(3.1)	1173	(3.4)	1105	(3.6)	< 0.001^c^
Chronic kidney disease	2707	(0.5)	178	(0.5)	160	(0.5)	0.45^c^
In vitro fertilization^g^	6904	(1.4)	706	(2.0)	675	(2.2)	< 0.001^c^
Presentation^h^							
Cephalic	454,795	(93)	31,640	(91)	27,636	(92)	
Non-cephalic	36,624	(8)	2960	(9)	2489	(9)	< 0.001^c^
Induction	42,894	(9)	4139	(12)	10,065	(33)	< 0.001^c^
Caesarean section^i^							
Elective	29,941	(6)	58	(0.2)	175	(0.6)	
Acute	33,704	(7)	4487	(13)	4427	(15)	
No	430,475	(87)	30,179	(87)	25,684	(85)	< 0.001^d^
Sex							
Male	251,018	(51)	18,348	(53)	15,876	(52)	
Female	243,863	(49)	16,411	(47)	14,456	(48)	< 0.001^c^
Small for gestational age	7140	(1.4)	367	(1.1)	407	(1.3)	< 0.001^c^
Apgar at 5 minutes^j^							
0–3	1493	(0.3)	126	(0.4)	95	(0.3)	
4–6	3103	(0.6)	404	(1.2)	384	(1.3)	
7–10	489,117	(99)	34,190	(99)	29,815	(98)	< 0.001^c^

^a^12–24 h between prelabor rupture of membranes (PROM) and delivery; ^b^ >  24 h between PROM and delivery

^c^ Linear-by-linear test for association

^d^ Kruskal-Wallis

^e^ Information on maternal age was missing in 21 children in the reference group, 3 children in the intermediate and in 3 children in the prolonged interval group

^f^ Information on Diabetes Mellitus was missing in 266 children in the reference group, 32 in the intermediate and 18 children in the prolonged interval group

^g^ Information on IVF was missing in 731 children in the reference group, 82 children in the intermediate and 63 children in the prolonged interval group

^h^ Information on Presentation was missing in 3462 children in the reference group, 159 children in the intermediate and 207 children in the prolonged interval group

^i^ Information on Caesarean section was missing in 761 children in the reference group, 35 children in the intermediate and 46 in the prolonged interval group

^j^ Information on Apgar was missing in 1168 children in the reference group, 39 children in the intermediate group and 38 children in the prolonged interval group

Mothers of children with CP were more likely to be nulliparous than mothers of children without CP (Table 2). Children with CP were more often small for gestational age at birth and were more likely to have low Apgar scores (Table 2).

**Table 2:** Maternal and infant characteristics according to whether or not the child was diagnosed with cerebral palsy

	Cerebral palsy				
	Yes		No		
	N	(%)	N	(%)	
Number of births	551	(100)	559,421	(100)	*P-value*
Maternal age^c^					
< 19	18	(3.3)	13,070	(2.3)	
20–34	421	(76)	454,117	(81)	
> 35	112	(20)	92,207	(17)	0.096^a^
Parity					
Para 0	270	(49)	226,537	(41)	
> 0 Para	281	(51)	332,884	(60)	< 0.001^b^
Any maternal diagnosis before pregnancy	245	(45)	232,001	(42)	0.15^b^
Any maternal diagnosis during pregnancy	177	(32)	164,254	(29)	0.16^b^
Diabetes mellitus^d^					
Diagnosed before pregnancy	4	(0.7)	3052	(0.5)	
Gestational	2	(0.4)	5120	(0.9)	0.34^b^
Thyroid disease	9	(1.6)	7839	(1.4)	0.64^b^
Recurrent urinary tract infection	15	(2.7)	17,721	(3.2)	0.55^b^
Chronic kidney disease	2	(0.4)	3043	(0.5)	0.56^b^
In vitro fertilization^e^	12	(2.2)	8272	(1.5)	0.17^b^
Presentation^f^					
Cephalic	493	(91)	513,578	(92)	
Abnormal	51	(9)	42,022	(8)	0.11^b^
Induction	90	(16)	68,197	(12)	0.003^b^
Caesarean section^g^					
Elective	23	(4)	30,151	(5)	
Acute	129	(23)	42,489	(8)	
No	399	(72)	485,939	(87)	< 0.001^b^
Sex					
Male	313	(57)	284,929	(51)	
Female	238	(43)	274,492	(49)	0.006^b^
Small for gestational age	33	(6)	7881	(1.4)	< 0.001^b^
Apgar after 5 minutes^h^					
0–3	52	(10)	1662	(0.3)	
4–6	61	(11)	3830	(0.7)	
7–10	435	(7)	552,687	(99)	< 0.001^a^

^a^ Linear-by-linear test for association

^b^ Pearson Chi-Square

^c^ Information on maternal age was missing in 27 of the children without CP

^d^ Information on diabetes mellitus was missing in 1 of the CP children, and 315 of the children without CP

^e^ Information on IVF was missing in 1 of the CP children and 875 of the children without CP

^f^ Information on presentation was missing in 7 of the CP children and 3821 of the children without CP

^g^ Information on Caesarean section was missing in 842 of the children without CP

^h^ Information on Apgar was missing in 3 of the CP children and 1242 of the children without CP

Stillbirths were more common in the reference group and decreased with increasing intervals between PROM and delivery (Table 3). In contrast, death during delivery and in the neonatal period did not differ significantly between the groups, although the observed prevalence rates were slightly higher for both of the exposed groups compared with the reference group (Table 3).

**Table 3:** Risk of stillbirth, death during delivery, neonatal mortality and cerebral palsy according to the time interval between prelabor rupture of membranes (PROM) and delivery

Number of births		Reference group			Intermediate interval (12–24 h)					Prolonged interval (>24 h)					
		N	Prevalence per 1000	95% CI	N	Prevalence per 1000	95% CI	OR^a^	95% CI^b^	N	Prevalence Per 1000	95% CI	OR	95% CI	*P-Value*^*c*^
		494,881			34,759					30,332					
Stillbirth	Yes	867	1.75	1.64–1.87	39	1.12	0.82–1.53	0.64	0.46–0.88	27	0.89	0.61–1.29	0.51	0.35–0.75	< 0.001
	No	494,014			34,720					30,305					
Death during delivery	Yes	63	0.13	0.10–0.16	8	0.23	0.12–0.45	1.81	0.87–3.77	5	0.16	0.07–0.39	1.29	0.52–3.22	0.247
	No	494,014			34,720					30,305					
Neonatal mortality	Yes	220	0.45	0.39–0.51	25	0.72	0.49–1.06	1.62	1.07–2.45	15	0.49	0.30–0.82	1.11	0.66–1.88	0.186
	No	493,794			34,695					30,290					
Cerebral palsy	Yes	467	0.94	0.86–1.03	38	1.09	0.80–1.50	1.16	0.83–1.61	46	1.52	1.14–2.02	1.61	1.19–2.18	0.002
	No	493,327			34,657					30,244					

^a^ OR; Odds ratio. ^b^ CI; Confidence interval. ^c^ Cochran-Armitage test for trend

A total of 551 children were diagnosed with CP (Table 3). There was an increasing prevalence of CP with increasing intervals between PROM and delivery (*p* = 0.002), although the OR for CP in the intermediate interval did not reach statistical significance (OR: 1.16; CI: 0.83 to 1.61). The OR for CP among children born after prolonged intervals was 1.61 (CI: 1.19 to 2.18) compared with children in the reference group (Table 3).

Adjusting for maternal age, parity, medical diagnoses before and during pregnancy, primary or gestational diabetes mellitus, thyroid disorders, recurrent urinary tract infection, parity, in vitro fertilization, sex, small for gestational age, fetal position, gestational age and birth weight did not change these findings essentially (Table 4).

**Table 4:** Crude and adjusted odds ratios (OR) with 95% confidence intervals (CI) for the association between the interval between rupture of membranes and delivery, and neonatal mortality and cerebral palsy

	Intermediate interval^a^ vs. reference group		Prolonged interval^b^ vs. reference group	
Neonatal mortality	OR	95% CI	OR	95% CI
Unadjusted	1.62	1.07–2.45	1.11	0.66–1.88
Adjusted for				
Maternal age	1.63	1.07–2.46	1.11	0.66–1.88
Parity	1.60	1.05–2.43	1.07	0.63–1.81
Any diagnosis before pregnancy	1.62	1.07–2.44	1.10	0.65–1.86
Any diagnosis during pregnancy	1.62	1.07–2.45	1.11	0.66–1..88
Thyroid disorder	1.62	1.07–2.45	1.11	0.66–1.87
Sex	1.61	1.06–2.44	1.11	0.66–1.87
Gestational age	1.60	1.06–2.42	1.10	0.65–1.85
Birth weight	1.60	1.06–2.42	1.07	0.63–1.80
Cerebral palsy				
Unadjusted	1.16	0.83–1.61	1.61	1.19–2.18
Adjusted for				
Maternal age	1.16	0.84–1.62	1.61	1.19–2.18
Parity	1.06	0.76–1.48	1.49	1.10–2.03
Any diagnosis before pregnancy	1.16	0.83–1.61	1.60	1.18–2.17
Any diagnosis during pregnancy	1.16	0.83–1.61	1.61	1.19–2.18
Thyroid disorder	1.16	0.83–1.61	1.61	1.19–2.18
Sex	1.15	0.83–.61	1.60	1.18–2.17
Gestational age	1.15	0.83–1.61	1.60	1.18–2.16
Birth weight	1.15	0.82–1.59	1.55	1.15–2.10

^a^12–24 h between prelabor rupture of membranes (PROM) and delivery; ^b^ > 24 h between PROM and delivery

Within the group of children with CP, there was no clear association between increasing intervals from PROM until delivery and CP-subtypes, gross- and fine motor function or associated problems (Table 5).

**Table 5:** Characteristics of children with cerebral palsy according to the time interval between PROM and delivery

	Reference group		Intermediate Interval^a^		Prolonged Interval^b^		
	N	(%)	N	(%)	N	(%)	*P-value*
Total number of children	467	(100)	38	(100)	46	(100)	
CP-subtype							
Spastic unilateral	246	(53)	18	(47)	22	(48)	
Spastic bilateral	147	(32)	13	(34)	20	(44)	
Dyskinetic	48	(10)	2	(5)	2	(4)	
Ataxic	24	(5)	3	(8)	2	(4)	
Non-classified CP	2	(0.4)	2	(5)	0	(0)	0.132^c^
GMFCS^f^							
I	267	(61)	19	(53)	23	(54)	
II	54	(12)	5	(14)	5	(12)	
III	20	(5)	0	(0)	2	(5)	
IV	26	(6)	4	(11)	7	(16)	
V	73	(17)	8	(22)	6	(14)	0.256^d^
MACS^g^							
I	157	(39)	14	(40)	15	(35)	
II	127	(32)	9	(26)	14	(33)	
III	32	(8)	3	(9)	3	(7)	
IV	19	(5)	3	(9)	4	(9)	
V	64	(16)	6	(17)	7	(16)	0.504^d^
Speech^h^							
I - Normal	226	(55)	16	(50)	18	(43)	
II - Imprecise	52	(13)	6	(19)]	7	(17)	
III – Unclear	56	(14)	4	(13)	7	(16)	
IV – Not understandable	76	(19)	6	(19)	10	(24)	0.200^d^
Epilepsy^i^	103	(25)	6	(19)	13	(33)	0.434^e^
Gastrostomy^j^	56	(13)	5	(15)	4	(9)	0.576^e^

^a^12–24 h between prelabor rupture of membranes (PROM) and delivery; ^b^ > 24 h between PROM and delivery. GMFC; Gross motor function classification system, MACS; Manual ability classification system

^c^ Kruskal-Wallis, exact Monte Carlo

^d^ Linear-by-linear test for association

^e^ Cochran-Armitage test for trend

^f^ Information missing on 27 children in the reference group, 2 children in the intermediate and 3 children in the prolonged interval group

^g^ Information missing on and 68 children in the reference group, 3 children in the intermediate and 3 children in the prolonged interval group

^h^ Information missing on 57 children in the reference group, 6 children in the intermediate and 4 children in the prolonged interval group

^i^ Information missing on 48 children in the reference group, 7 children in the intermediate and 6 children in the prolonged interval group

^j^ Information missing on 44 children in the reference group, 5 children in the intermediate and 3 children in the prolonged interval group

Descriptions of cerebral MRI scans were available for 434 (79%) of the children with CP. The median age when the scans were performed was 16 months (interquartile range 2 to 34 months). The proportion of children with basal ganglia pathology and diffuse cortical injury increased with increasing intervals from PROM to delivery, while the proportion of children with white matter injury of immaturity decreased and was highest in the reference group (Table 6).

**Table 6:** Cerebral MRI-findings in children with CP according to the time interval between PROM and delivery

	Reference group		Intermediate interval^a^		Prolonged interval^b^		
	N	(%)	N	(%)	N	(%)	
Children examined with MRI^c^	366	(100)	28	(100)	40	(100)	*P*-value^d^
Normal	66	(18)	5	(18)	3	(8)	0.120
Basal ganglia pathology	65	(18)	8	(29)	13	(33)	0.013
White matter injury	158	(43)	11	(39)	10	(25)	0.030
Focal cortical injury	67	(18)	4	(14)	10	(25)	0.437
Diffuse cortical injury	58	(16)	7	(25)	11	(28)	0.037
Corpus callosum pathology	48	(13)	3	(11)	10	(25)	0.076
Cerebellum pathology	19	(5)	0	(0)	1	(3)	0.263

^a^ 12–24 h between prelabor rupture of membranes (PROM) and delivery; ^b^ > 24 h between PROM and delivery

^c^ 434 of 551 children with CP (79%) had done a Cerebral MRI

^d^ Cochran-Armitage test for trend

## Disscussion

### Main findings

In this population based study, we found that the risk for CP increased with increasing intervals between PROM and delivery of the child. An interval of more than 24 h was associated with a 60% excess risk for CP compared with the reference group, independent of other antenatal risk factors for CP. However, the absolute risk for CP was low and there was no excess risk for death during delivery or in the neonatal period.

Among children with CP, there was no association between increasing intervals between PROM and delivery and CP subtypes or severity of impairments. However, among those who had been examined with cerebral MRI, the proportion with findings consistent with hypoxic-ischemic injuries (i.e. diffuse cortical injury and basal ganglia pathology) increased with increasing intervals, while the proportion consistent with white matter lesions, usually a sequelae of injuries in mid-pregnancy, decreased.

### Strengths and limitations

Strengths of the present study are the large number of births and the prospective recording of data in the MBRN and the CPRN. The percentage of children with PROM (11.6%) is comparable to what has been reported in the literature (6–19%) [1]. The main findings are unlikely to be caused by chance as indicated by the low *P*-values. The correctness of the CP diagnosis in the CPRN at five years of age, and the unbiased selection of cases as documented in a validation study are further strengths [23]. According to updated information from the register, 83% of all children with CP born during in Norway during 1999–2009 were registered in the CPRN [24]. The corresponding misclassification of approximately 100 children with CP as controls is negligible compared with the 500,000 children comprising the latter group.

Although the exact timing of the membrane rupture, the onset on contractions, and the delivery is recorded in medical records of obstetric departments, this information is categorized by the MBRN as described in the methods section. This categorization implies that an interval of less than 12 h between PROM and delivery is included in the reference group. This lack of detailed information leads to some misclassification. In addition, some children in the reference group may have been born more than 24 h after rupture of membranes, even if the rupture occurred at or after onset of contractions. However, these limitations have most likely diluted the differences between the groups.

In the study period, according to Norwegian guidelines, intrapartum antibiotic prophylaxis was recommended to reduce the risk of GBS disease in the newborn if “there were signs of infection or maternal fever intrapartum (prolonged rupture of membranes, foul smelling amniotic fluid, fetal tachycardia, increasing C-reactive protein).” [25] Thus, intrapartum antibiotic prophylaxis was most likely not administered in deliveries where a prolonged interval from PROM to delivery was the only risk factor. The lack of information on maternal antibiotic treatment and suspected maternal infection (i.e. fever, or clinical chorioamnionitis) around delivery, in particular maternal and infant GBS-status (routine screening for maternal GBS colonization is not performed in Norway), is another limitation of the study. However, although the data prevents us from speculating on infectious etiology, we consider it most unlikely that the increased risk for CP associated with a prolonged interval is explained solely by a more restricted use of intrapartum antibiotic prophylaxis in Norway than in some other countries.

The cerebral MRI images were interpreted by different radiologists at different hospitals, which is another limitation. The reliability of these descriptions is therefore uncertain. Moreover, MRI examinations were completed at different ages of the children. Thus, caution is needed in the interpretation of these results, although they may be considered plausible.

The study was restricted to term born singletons without congenital malformations to exclude confounding by prematurity, multiple births and malformations. In particular, the management of PROM in preterm deliveries is likely to be significantly different from the management of PROM in the otherwise uncomplicated pregnancy at term. Furthermore, multivariable analyses did not suggest confounding by the available antenatal, known risk factors for CP.

### Comparison to literature and interpretation of findings

Few studies have addressed the potential association between increasing intervals from PROM to delivery and CP. In a systematic review on risk factors for CP, McIntyre et al. identified one study in term born children [10]. That study found a non-significant increased risk for CP following an interval between PROM and delivery of more than 24 h (OR 1.54, CI 0.37–6.27) [11]. Albeit non-significant, the size effect was similar to the one we found, and the lack of statistical difference was probably due to a limited sample size (101 cases and 308 controls).

We managed to identify two more studies on the association between prolonged intervals from PROM until delivery, and CP. However, both studies included preterm children, and are therefore not comparable to our study [26, 27].

Our findings may be compared with three studies on the association between a long interval from rupture of membrane to delivery and CP, although these studies did not study PROM. The sample sizes of these studies were smaller than in our study, but the reported effect sizes were comparable to our findings (i.e. OR 1.4–2.4) [28–30], however the results were not statistically significant.

The association between a prolonged interval from PROM to delivery and CP could be caused by a perinatal infection. While our study does not provide data supporting a causal relationship between a prolonged interval, infections and CP, other studies have found that a prolonged interval increases the risk for perinatal infections [8], and that perinatal infections increase the risk of CP [9].

However, it may be noteworthy that in the group of children with CP, the proportion with diffuse cortical injury and basal ganglia pathology on cerebral MRI increased with increasing intervals from PROM to delivery. These findings are characteristic of hypoxic-ischemic injuries at term [31–33], and they are also highly predictive of CP [34]. We therefore speculate that a long interval between PROM and delivery may increase the vulnerability of the neonatal brain, lowering the threshold at which a reduction in blood supply to the fetus may lead to perinatal hypoxic-ischemic injury. This vulnerability might be caused by an infection and/or inflammation. Moreover, the decreasing proportion of white matter lesions with increasing intervals from PROM to delivery may lend further support to this speculation, since such findings are likely to suggest prolonged partial asphyxia [35], and are characteristic of injuries occurring earlier in pregnancy. On the other hand, 10 children with CP in the prolonged interval-group had white matter injury, the typical lesion in children with CP born preterm. In these children it is possible that the brain injury predated PROM.

Another possible explanation of our findings could be reverse causality, i.e. that the birth process is slower in fetuses with an antenatal brain injury. However, we consider this less likely, based upon the cerebral MRI findings, and the exclusion of congenital malformations and plurals.

The decreasing prevalence of stillbirths with increasing intervals between PROM and delivery can most likely be explained by reverse causality as antenatal stillbirths are likely to be induced early.

## Conclusion

The findings of the present study provide some evidence that may be useful in the discussion with the mother regarding the choice between immediate or expectant delivery when PROM is present. First of all, a prolonged interval between PROM and delivery was not associated with increased perinatal or neonatal mortality. This may be reassuring for those advocating expectant management. On the other hand, we found that a prolonged interval was associated with a 60% excess risk for CP. However, this risk must be weighed against the very low absolute risk of CP. The design of the present study does not allow recommendations for interventions to reduce the excess risk of CP associated with a prolonged interval. Further studies are therefore needed to investigate whether a reduction in relative risk for CP following PROM can be achieved through more active interventions, without increasing the risk of other adverse outcomes.

## Data Availability

The data was originally obtained from The Medical Birth Registry of Norway (MBRN) and The Cerebral Palsy Register of Norway (CPRN). Permissions to access the data were obtained from the CPRN, MBRN, and the Regional Committees for Medical and Health Research Ethics (REC). A request of access to anonymized data by external researches will have to be approved by the two registers as well as by the REC, and be in line with the EU General Data Protection Regulations (GDPR). Inquiries may be sent to the authors or to the Head of the Cerebral Palsy Register of Norway, Vestfold Hospital Trust, PB 2168, 3103 Tønsberg, Norway.

## Abbreviations

CP: Cerebral Palsy
CPRN: The Cerebral Palsy Register of Norway
GA: Gestational age
MBRN: The Medical Birth Registry of Norway
MRI: Magnetic resonance imaging
PROM: Prelabor rupture of membranes
